# PD-1/PD-L1 Pathway Modulates Macrophage Susceptibility to *Mycobacterium tuberculosis* Specific CD8^+^ T cell Induced Death

**DOI:** 10.1038/s41598-018-36403-2

**Published:** 2019-01-17

**Authors:** Guadalupe Verónica Suarez, Claudia del Carmen Melucci Ganzarain, María Belén Vecchione, César Ariel Trifone, José Luis Marín Franco, Melanie Genoula, Eduardo José Moraña, Luciana Balboa, Maria Florencia Quiroga

**Affiliations:** 1 Consejo Nacional de Investigaciones Científicas y Técnicas (CONICET)-Universidad de Buenos Aires. Instituto de Investigaciones Biomédicas en Retrovirus y Sida (INBIRS), Buenos Aires, Argentina; 2 Consejo Nacional de Investigaciones Científicas y Técnicas (CONICET)-Instituto de Medicina Experimental (IMEX)-Academia Nacional de Medicina, Buenos Aires, Argentina; 3 Instituto de Tisioneumonologia Dr R. F. Vaccarezza, Universidad de Buenos Aires, Buenos Aires, Argentina

## Abstract

CD8^+^T cells contribute to tuberculosis (TB) infection control by inducing death of infected macrophages. *Mycobacterium tuberculosis* (*Mtb*) infection is associated with increased PD-1/PD-L1 expression and alternative activation of macrophages. We aimed to study the role of PD-1 pathway and macrophage polarization on *Mtb*-specific CD8^+^T cell-induced macrophage death. We observed that both PD-L1 on CD14^+^ cells and PD-1 on CD8^+^T cells were highly expressed at the site of infection in pleurisy TB patients’ effusion samples (PEMC). Moreover, a significant increase in CD8^+^T cells’ *Mtb*-specific degranulation from TB-PEMC vs. TB-PBMC was observed, which correlated with PD-1 and PDL-1 expression. In an *in vitro* model, M1 macrophages were more susceptible to *Mtb*-specific CD8^+^T cells’ cytotoxicity compared to M2a macrophages and involved the transfer of cytolytic effector molecules from CD8^+^T lymphocytes to target cells. Additionally, PD-L1 blocking significantly increased the *in vitro* Ag-specific CD8^+^T cell cytotoxicity against IFN-γ-activated macrophages but had no effect over cytotoxicity on IL-4 or IL-10-activated macrophages. Interestingly, PD-L1 blocking enhanced *Mtb*-specific CD8^+^ T cell killing of CD14^+^ cells from human tuberculous pleural effusion samples. Our data indicate that PD-1/PD-L1 pathway modulates antigen-specific cytotoxicity against M1 targets *in-vitro* and encourage the exploration of checkpoint blockade as new adjuvant for TB therapies.

## Introduction

*Mycobacterium tuberculosis* (*M*. *tuberculosis*, *Mtb*) has the ability to manipulate cell death pathways in infected macrophages, which constitutes a virulence mechanism for this pathogen^1^. While virulent strains of *M*. *tuberculosis* induce necrosis of infected macrophages and actively inhibit the induction of macrophage apoptosis, attenuated *M*. *tuberculosis* strains generally do not. Additionally, cumulative evidence shows that apoptosis results in lower viability of *M*. *tuberculosis* induced pharmacologically either by the pathogen itself or by cytotoxic lymphocytes^2^. Also, apoptotic infected macrophages release less viable bacilli than necrotic macrophages^3^, which leads to the hypothesis that infected cell necrosis may be exploited by *M*. *tuberculosis* to propagate across the infected organism. On the other hand, while intracellular milieu of infected macrophages is completely modified by *M*. *tuberculosis* in its own favor leading to the establishment of an innocuous microenvironment to the bacteria, phagocytosis of apoptotic bodies containing viable bacteria by uninfected macrophages can lead to the definitive death of the bacilli in a process called efferocytosis^4^. This mechanism not only contributes to bacterial clearance but also it is fundamental to *M*. *tuberculosis* antigens presentation by dendritic cells to naïve CD8^+^ T cells, contributing to the start and preservation of CD8^+^ T cell responses against the pathogen^4^.

Evidence pointing to an essential role of CD8^+^ T cells during *M*. *tuberculosis* infection in humans is scarce. In this sense, the relevance of cytotoxic anti-tubercular immune responses have been highlighted in humans, since it has been reported that anti-TNF-α blocking antibodies administration leads to the elimination of a terminally-differentiated CD8^+^ T cell population in rheumatoid arthritis patients with latent tuberculosis infection. This is thought to be partly responsible for their increased predisposition to TB reactivation^5^. Also, recent evidence suggests that CD8^+^ T cells contribute to the optimal control of *M*. *tuberculosis* infection through several effector mechanisms, including the induction of infected-macrophage apoptosis (i.e., cytotoxicity)^6,7^. Finally, we have already described a deficient CD8^+^T cell differentiation in the context of HIV-TB co-infection, which has an impact on cell functionality^8^.

*M*. *tuberculosis* control relies fundamentally on bactericidal mechanisms induced by the activation of infected macrophages. Furthermore, macrophage activation is heterogeneous, and it is divided into three different profiles: M1 macrophages, which are differentiated in response to type 1 cytokines (like IFN-γ) and microbial products; M2a macrophages are induced by type 2 cytokines (like IL-4 or IL-13) and M2b/c macrophages are induced by regulatory signals (like IL-10 or immune complexes)^9^. Previously, it was demonstrated that M1 polarization of macrophages is critical for *M*. *tuberculosis* control, with M1 macrophages promoting granuloma formation and macrophage bactericidal activity, and M2-polarized macrophages inhibiting these effects^10^. In this regard, it has been shown that the *E*arly *S*ecreted *A*ntigenic *T*arget 6 kDa or ESAT-6, a pathogenicity mycobacterial factor, has the potential to switch human macrophages from an M1 to an M2 phenotype^11^. Also in mice, the avirulent strain H37Ra induces M1-related molecules of *M*. *tuberculosis* infected macrophages, whereas its virulent counterpart H37Rv induces an M2-phenotype, highlighting the relevance of mycobacterial virulence factors on macrophage function^12^. Conversely, IL-4 activation of macrophages deprives them of the control mechanisms to limit mycobacterial growth, allowing its persistence within infected macrophages^13^.

Although the role of macrophage activation in *M*. *tuberculosis* control is well established^14,15^, the consequences of macrophage polarization on their susceptibility to CD8^+^ T cell-killing machinery have been poorly explored. Furthermore, the relevance of inhibitory checkpoints in this cellular interaction (i.e., the interaction between CD8^+^ T lymphocytes and polarized macrophages) is a completely unexplored issue, even outside the field of human infections.

The role of the PD-1/PD-L pathway, which is fundamental in T cell biology^16^, is controversial in the context of *M*. *tuberculosis* infection. Considering other diseases, it was shown that the PD-1/PDL pathway is an important checkpoint in cancer immunotherapy, since the inhibition of this pathway enhances tumor-specific CD8^+^ T-cell responses^17–19^. Moreover, a novel therapeutic strategy aimed at blocking the PD-1 expression on human antigen-specific cytotoxic T-lymphocytes has been described based on CRISPR-the Cas9 genome editing^20^. In human tuberculosis, while some authors demonstrated that the induction of PD-1 expression during infection is detrimental as it inhibits protective adaptive immune responses^21,22^, others have shown that its induction is necessary to inhibit the exacerbated immune response that leads to tissue damage during active infection^23,24^. Yet, the role of this pathway on the regulation of the CD8^+^ T cell function during *M*. *tuberculosis* infection has not been studied thoroughly^25^.

In this context, the data presented here shows that while M1 macrophages are more susceptible to antigen-specific CD8^+^ T cell killing, the greater expression of PDL-1 on M1 target cells counteracts the activation of CD8^+^ T cells, inhibiting macrophage killing by cytotoxic effectors. We also demonstrate that PD-1 and PDL-1 are highly expressed at the site of infection during human tuberculosis and that these molecules are involved in *Mtb*-specific cytotoxic killing of CD14^+^ targets cells from human pleura effusion (PE) samples. Altogether, our results encourage the exploration of CD8-targeted anti-PD-1 blockade as a new adjuvant therapy for tuberculosis treatment.

## Methods

### TB-pleurisy patients

Pleural effusion (PE) and paired peripheral blood (PB) samples were collected from HIV-negative TB pleurisy patients following standard procedures for diagnosis purposes at the Hospital de Infecciosas “Dr. Francisco Javier Muñiz”, Buenos Aires, Argentina. The research was carried out in accordance with the World Medical Association Declaration of Helsinki (2013), and approved by the Ethics Committees of the Hospital “Francisco J. Muñiz” and the Academia Nacional de Medicina de Buenos Aires (protocol number: NIN-2612-17). Written informed consents were obtained from all participants before sample collection. The diagnosis of tuberculous pleurisy was based on a positive Ziehl–Nielsen stain or Lowestein–Jensen culture from PE and/or histopathology of pleural biopsy, and was further confirmed by an *M*. *tuberculosis*-induced IFN-γ response and an ADA-positive test^26^. None of the patients had multidrug-resistant TB or received anti-tuberculosis treatment before sample collection. The male/female group distribution was 18/4 and the median age was 39 years, interquartile range (IQR) 18–75 years. In addition to TB pleurisy patients, 13 individuals latently infected with *M*. *tuberculosis* were enrolled (male/female distribution 3/2, median age 42 years, IQR 25–85 years). The entire group of individuals were BCG (Bacillus Calmette-Guerin)-vaccinated at birth. Mononuclear cell were isolated from pleural effusion (PEMC) and blood samples (PBMC) by Fycoll-Paque PLUS® gradient (GE Healthcare Life Sciences). Then, PBMC or PEMC were stained by PD-L1 PE, CD8 APC or PerCP, CD3 FITC, CD14 APC, PD-1 PE (BD Biosciences), CD27 APC, CD45RA PE-Cy7 (Biolegend) and NearER Live Dead reagent (Invitrogen) and analyzed in a FACSCanto flow cytometer (BD Bioscences). In another set of experiments, cytotoxic degranulation of CD8^+^ T cells was assessed by CD107a/b expression (20 μL/mL; BD Biosciences) after culture in the presence of *M*. *tuberculosis*, as described by our group and elsewhere^8,22,27^. Data was analyzed using the FlowJo® v10.0.8 software (FlowJo, LLC).

### *In vitro* differentiation and polarization of macrophages

Monocytes were purified from PBMC obtained from 30 blood buffy-coats (provided by the Hemotherapy Division, Sanatorio “Dr. Julio Mendez”, Buenos Aires, Argentina), by Fycoll-Paque PLUS® gradient followed by Percoll® gradient (GE Healthcare Life Sciences). All healthy donors were between 18–65 year old; completed and passed a survey on blood donation; and were screened for serological markers of HIV, HCV, HBV, HTLV-I/II, Syphilis, Chagas disease, and Brucellosis, before being enrolled. Cell purity was evaluated by flow cytometry through forward *vs*. side scatter plots. Samples with purity lower than 60% of monocytes were discarded. Monocytes were then plated at 1 × 10^6^ cells/ml in 24 well culture plates using FBS-free RPMI 1640 (Sigma-Aldrich). Adhesion was achieved by incubating cells for 1 h at 37 °C. After washing cells with PBS, adherent cells were cultured in RPMI 1640 (Sigma-Aldrich) supplemented with 10% FBS (Gibco, Thermo Fisher Scientific), L-glutamine (2 mM), penicillin (100 U/ml), streptomycin (100 μg/ml, Gibco, Thermo Fisher Scientific) and GM-CSF (30 ng/ml, Miltenyi Biotec). After 5 days, in order to induce macrophage polarization, macrophages were detached and re-plated in the presence of the following cytokine cocktails: GM-CSF (30 ng/ml) plus IFN-γ (M1, 500 UI/ml, BEI resources, NR-3086), IL-4 (M2a, 10 ng/ml, Miltenyi Biotec) or IL-10 (M2c, 10 ng/ml, Miltenyi Biotec) for 2 additional days. Non-polarized macrophages (cultured in the presence of GM-CSF) were used as control in each experiment. Also, macrophages from different donors were polarized in each independent experiment.

The day of the experiment, macrophage phenotype was evaluated by assessing several markers by flow cytometry, as reported previously^28^. Data were analyzed with FlowJo Software (Version 10.4, Tree Star) after gating on the myeloid population in the FSC/SSC window and excluding cell aggregates (doblets) by FSC-A/FSC-H. To normalize the results from independent experiments, values were depicted as the ratio of the geometric mean fluorescence intensity (MFI) of the marker of interest over the MFI of the corresponding isotype control.

### *In vitro* cytotoxicity assays

#### Preparation of effector CD8^+^ T cells

PBMC from buffy coats were isolated by Fycoll-Paque PLUS® gradient and cultured at 15 × 10^6^ cells per well in 5 ml of complete RPMI (RPMI 1640 -Sigma-Aldrich- supplemented with 10% FBS -Gibco, Thermo Fisher Scientific-, L-glutamine −2 mM-, penicillin −100 U/ml-, streptomycin −100 μg/ml, Gibco, Thermo Fisher Scientific-) in 6 multi-well plates and cultured in the presence of *M*. *tuberculosis* (10 μg/ml, equivalent to a bacillus/cell ratio = 0.25 of *Mycobacterium tuberculosis*, Strain H37Rv, Gamma Irradiated Whole Cells, NR-14819 BEI Resources, NIAID, NIH) or Cytomegalovirus, Epstein Barr and Influenza (CEF) Control Peptide Pool (1 ng/ml, NIH AIDS Reagent Program, Division of AIDS, NIAID, NIH) plus IL-2 (100 ng/ml) for 7 days at 37 °C in a humidified atmosphere rich in CO_2_. Afterwards, CD8^+^ T cells were washed, isolated performing a negative selection protocol, as indicated by the manufacturer (CD8^+^ T cell isolation kit, Miltenyi Biotec), re-suspended in fresh culture media and proceeded to perform the cytotoxicity assays.

#### Preparation of target cells and cytotoxicity assay

The day of the experiment, polarized macrophages were loaded with pre-titrated *M*. *tuberculosis* (10 μg/ml, equivalent to a bacillus/cell ratio = 0.25) for 2 h, CEF (5 ng/ml) for 1 h or anti-CD3 antibodies (5 μg/ml) for 10′, washed and co-cultured with autologous isolated CD8^+^ T cells. CD8^+^ T cells were previously stimulated with *M*. *tuberculosis* or CEF as described above and boosted with phorbol-12-miristate-13-acetate (PMA) and Ionomycin (5 ng/ml and 500 ng/ml respectively, Sigma-Aldrich) for 10’ and washed extensively. Unless indicated, experiments were performed at a final CD8^+^ T cell: macrophage ratio of 2:1 in 200 μl of complete RPMI for 18 h.

After co-culture, adherent and non-adherent cells were collected by detachment with a trypsin solution (TrypLE™ Express, Thermo Fisher Scientific), washed and immediately incubated for 10′ with 7-amino-actinomycin D solution (7-AAD, Thermo Fisher Scientific) as indicated by the manufacturer. Then, cells were analyzed by flow cytometry in a FACSCanto (BD Biosciences) flow cytometer. Data was analyzed with FlowJo® v10.0.8 software (FlowJo, LLC). In a separate set of experiments, pleural effusion target cells were isolated by positive selection (CD14 Microbeads, Miltenyi Biotec) and CD8^+^ cells were isolated by negative selection (CD8^+^ T Cell Isolation Kit, Miltenyi Biotec). Afterwards, cytotoxicity assay was performed as indicated above.

### Statistical analysis

Statistical analyses were conducted using GraphPad Prism software version 5.01. Comparisons between two groups were evaluated by the Wilcoxon test for paired samples. Alternatively, comparisons among three or more groups were done using the Kruskal–Wallis analysis of variance, followed by post-hoc comparisons (Dunns), or using the ANOVA test followed by the Bonferroni test, when applicable. For paired samples, Friedman test was performed. Correlation analyses were determined using the Spearman’s rank test. Statistical analysis and display of multicomponent distributions were performed by partial permutation test using SPICE v5.1 (http://exon.niaid.nih.gov/spice/). For all statistical comparisons, a *p* value < 0.05 was considered significant.

## Results

### Up-regulation of PD-1 pathway molecule expression on cells from TB pleurisy patients

**Figure 1:**
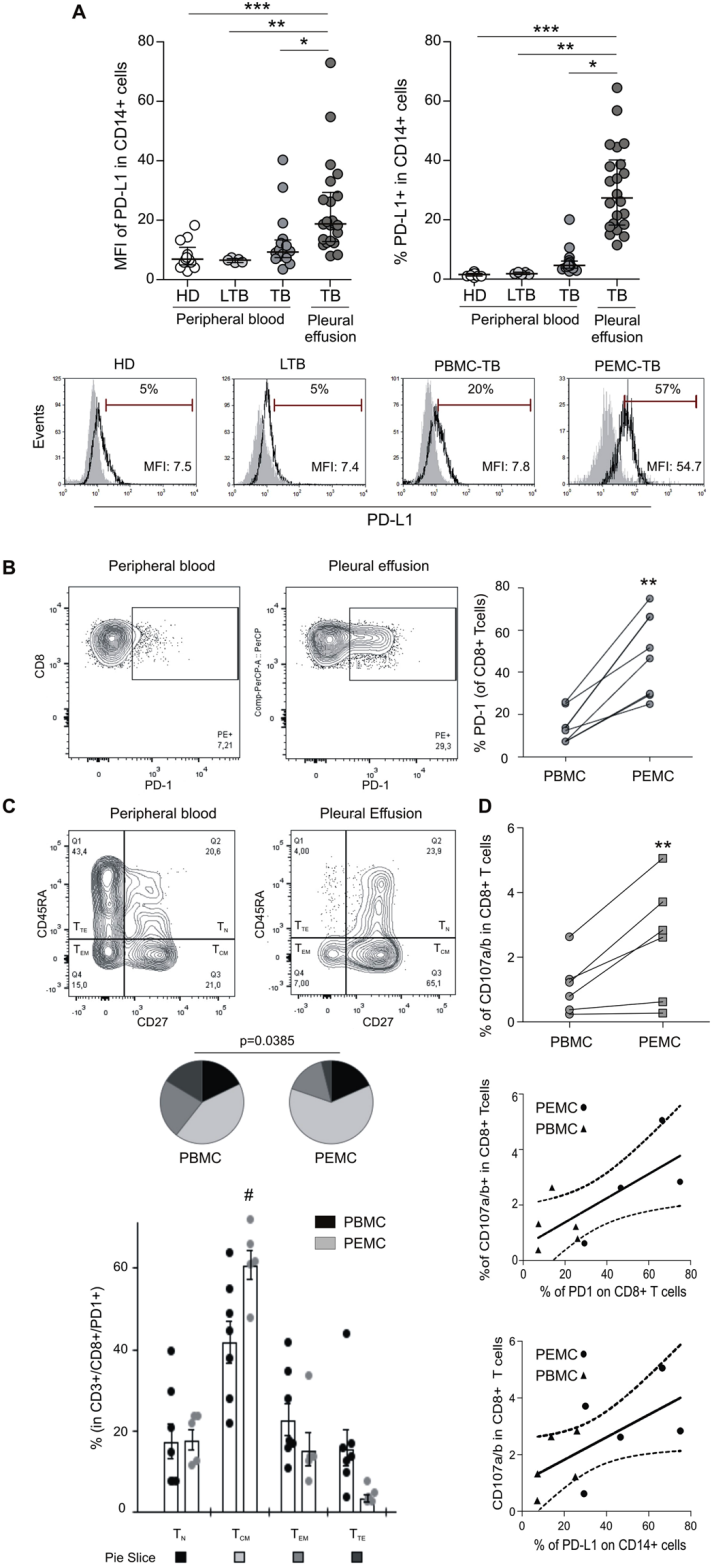
Expression of PD-1 and PD-L1 in cells from pleurisy TB patients’ pleural effusions. (**A**) Upper panel. MFI of PD-L1 (left) and % of PD-L1^+^ cells (right) in CD14^+^ cells from peripheral blood or pleural effusions from pleurisy TB patients (TB), peripheral blood from latent TB infected individuals (LTB) or healthy donors (HD). Each symbol represents an individual subject. Horizontal lines represent median values and whiskers represent interquartile range. p < 0.05; **p < 0.01; ***p < 0.001 by ANOVA followed by Dunn’s posttest. Lower panel. Flow cytometry histograms depicting PDL-1 expression of CD14^+^ cells from representative individuals among each group. (**B**) PD-1 expression on PEMC or PBMC CD8^+^ T cells from TB patients. Horizontal lines connect data from each individual (right panel). A representative cytogram from peripheral blood (left panel) or pleural effusion mononuclear cells (center) is shown. **p < 0.01, Wilcoxon Signed Rank Test. (**C**) Representative cytograms depicting CD27 and CD45RA expression on CD3^+^/CD8^+^/PD-1^+^ cells from peripheral blood (left) or pleural effusion (right) TB samples. Bottom: pie charts summarize the data and each slice corresponds to the mean proportion of CD3^+^/CD8^+^/PD-1^+^ cells for each phenotype. Bar graph represents possible phenotypes, which are shown on the x-axis whereas percentages of distinct T-cell subsets within CD3^+^/CD8^+^/PD-1^+^ cells are shown on the y-axis. Horizontal lines represent the median range and each point represents an individual subject; asterisks indicate a significant difference between groups; #p < 0.05. Comparisons of phenotype distribution were performed using the partial permutation test as described in ref.^32^ and the Kruskal–Wallis test followed by Dunn’s multiple comparisons posttest. (**D**) Upper panel: CD107a/b expression in CD8^+^ T cells after *Mtb*-stimulation of PBMC or PEMC from TB patients. Horizontal lines connect data from each individual. **, p < 0.01, non-parametric student *t* Test for paired samples. Middle: Correlation analysis between PD-1 expression on CD8^+^ T cells and CD107a/b in CD8^+^ T cells from TB PEMC and PBMC. p = 0.0287, r^2^ = 0.5183. Lower panel: Correlation analysis between the %PD-L1 expression on CD14^+^ cells and CD107a/b in CD8^+^ T cells from TB PEMC and PBMC. p = 0.0477, r^2^ = 0.4056.

The role of PD-1/PDLs as negative regulators of T-cell effector functions has been extensively studied^21,24,29^. Also, the study of cytotoxic T-cells in tuberculosis is an issue of increasing relevance, because CD8^+^ T cells not only produce IFN-γ, a macrophage-activating cytokine^30^, but also are able to kill infected macrophages^5^. In this context, we aimed to assess the expression of PD-1 pathway receptors on peripheral blood mononuclear cells (PBMC) and TB pleural effusion CD8^+^T cells and CD14^+^ cells. Thus, we observed that the expression of PD-L1 in CD14^+^ cells from pleural effusion mononuclear cells (PEMC) was highly increased compared to PBMC CD14^+^ cells from TB patients and also to peripheral CD14^+^ cells from individuals with latent TB or healthy donors, both in terms of the expression level on a per-cell basis or the percentage of positive cells (Fig. 1A). On the other hand, when analyzing the phenotype of CD8^+^ T cells, we observed that the PD-1 expression was significantly higher in CD8^+^ T cells from PEMC compared to paired peripheral CD8^+^ T cells from TB patients (Fig. 1B). T cells were phenotypically discriminated in terms of CD27/CD45RA expression by flow cytometry as naïve (T_N_), central memory (T_CM_), effector memory (T_EM_), and terminal effector (T_TE_) T lymphocytes, as described by us previously^31^. Thus, partial permutation test^32^ evinced that, in contrast to peripheral blood cells, CD8^+^ PD-1^+^ T cells from pleural effusion exhibited a distinct effector/memory population distribution (Fig. 1C, pies) with a predominant central memory phenotype (*p* = 0.05 comparing T_CM_ between PD-1^+^ PBMC and PD-1^+^ PEMC, Fig. 1C, Bars), therefore suggesting an exhausted phenotype in the pleural compartment^8,33^. Moreover, the observation of a memory exhausted phenotype in the PD-1 compartment was corroborated by comparing PD-1^+^ and bulk CD8^+^ T cells, which revealed a significantly dissimilar effector/memory distribution between both populations in peripheral blood and also in the pleural compartment (*p* = 0.0053 and *p* = 0.0040, respectively by partial permutation test, Supplementary Fig. 1). In the PE context, these differences were due to diminished naïve and terminal effector cells and higher central memory T cells in PD-1^+^ cells (*p* = 0.006 comparing T_N_ between PE-PD-1^+^ and PE-T CD8^+^; *p* = 0.006 comparing T_CM_ in PE-PD-1^+^ and PE-T CD8^+^ and *p* = 0.045 comparing T_TE_ in PE-PD-1^+^ and PE-T CD8^+^). In contrast, in peripheral blood those differences were due to lower naïve cells and higher proportions of central memory and effector memory cells (*p* = 0.048 comparing T_N_ between PB-PD-1^+^ and PB-T CD8^+^; *p* = 0.041 comparing T_EM_ in PB-PD-1^+^ and PB-T CD8^+^ and *p* = 0.002 comparing T_CM_ in PB-PD-1^+^ and PB-T CD8^+^, Supplementary Fig. 1A). Interestingly, in addition to a significant increase in cytotoxic *Mtb*-specific degranulation by CD8^+^ T cells from TB-PEMC *vs*. TB-PBMC (measured as CD107a/b^+^ CD8^+^ T cells after *Mtb* stimulation, Fig. 1D -upper panel-), a positive correlation was observed between PD-1 expression on CD8^+^ T cells or PDL-1 expression on CD14^+^ cells, and the cytotoxic capacity of CD8^+^ T cells (Fig. 1D, middle and lower panel, respectively). Finally, since both PD-1 and PDL-1 expression levels are regulated by IFN-γ^34^ we assessed this cytokine’s concentration and observed higher IFN-γ concentrations in pleural effusions compared to peripheral blood (Supplementary Fig. 1B). Overall, these results show an increase in both PD-1 and PD-L1 expressions, higher IFN-γ concentrations and increased CD8-mediated cytotoxicity at the site of infection during TB pleurisy.

### Macrophage susceptibility to antigen-specific CD8^+^ T cell mediated cytotoxicity is related to macrophage-polarization state

**Figure 2:**
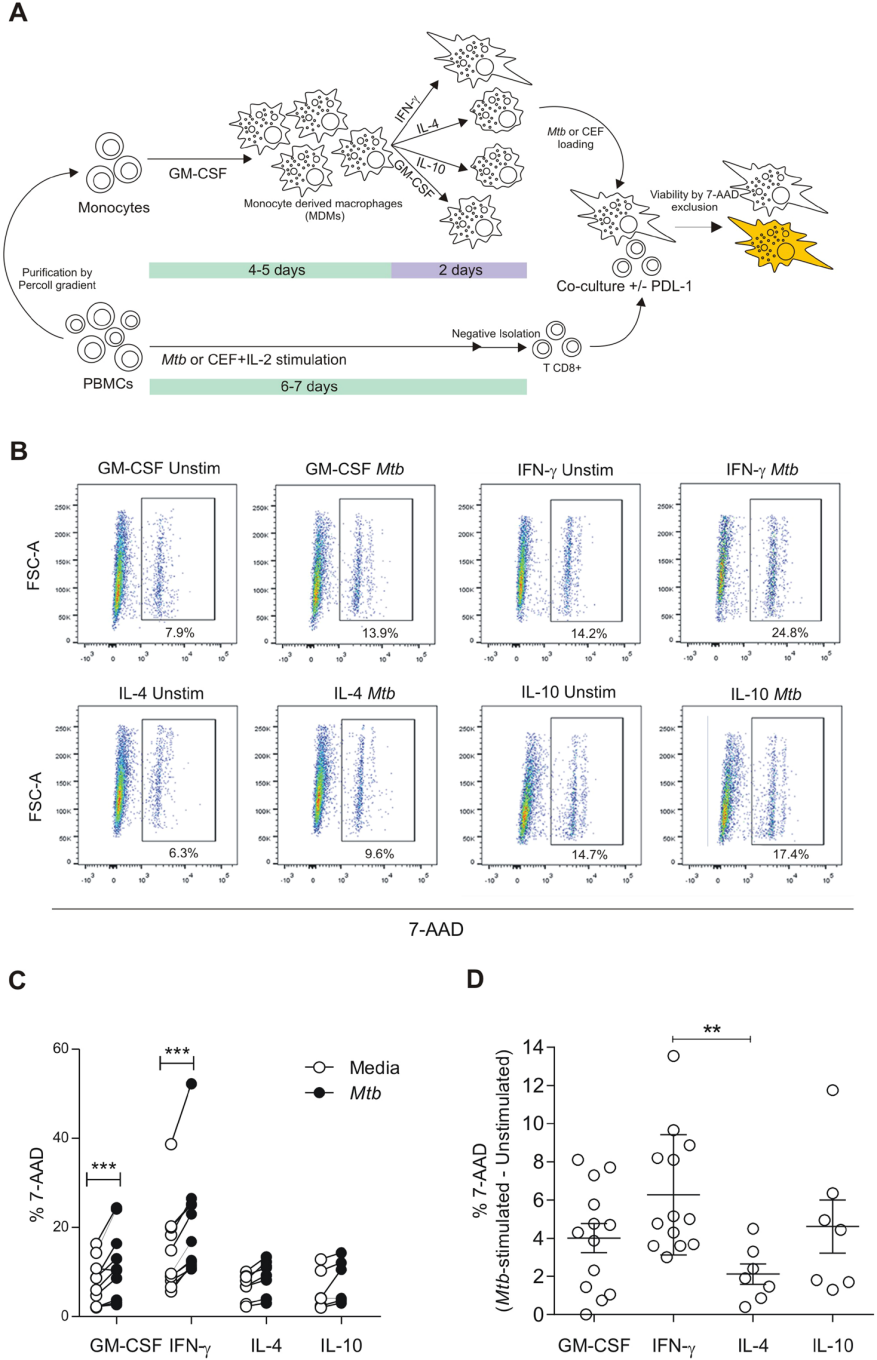
Effect of macrophage polarization on *in vitro* CD8^+^ T cell-mediated cytotoxicity. (**A**) Graphical description of the cytotoxicity assay. Monocyte derived macrophages were differentiated from peripheral blood by culturing monocytes with GM-CSF (30 ng/ml) for 6–7 days alone or supplemented with IFN-γ (500 U/ml), IL-4 (10 ng/ml) or IL-10 (10 ng/ml) for 2 additional days. Then, macrophages were cultured with *M*. *tuberculosis* (10 μg/ml) or CEF (1 ng/ml) as described in *Methods*. After that, macrophages were co-cultured for 18 h with syngeneic CD8^+^ T cells previously expanded with *Mtb* or CEF + IL-2 as described in *Methods*. Finally, macrophage death was determined by 7-AAD incorporation by flow cytometry. (**B**) FSC-A vs. 7-AAD dot plots of representative co-cultures of CD8^+^ T cells and macrophages (2:1 ratio) loaded with *Mtb*. (**C**) Figure shows the percentage of 7-AAD^+^ macrophages from 13 independent experiments, each one including one donor assayed by duplicate. Horizontal lines connect data from media and *Mtb*-stimulated macrophages cultured under each polarizing condition. ***p < 0.001 by ANOVA followed by Bonferroni post-test. (**D**) Comparison of CD8-induced macrophage death between polarizing conditions. Media values were subtracted and negative values were set to 0. **p < 0.01 by ANOVA followed by Bonferroni posttest.

It has been described that *Mtb* infection could modulate macrophage polarization from an M1 profile to an M2-like one^11,12^. However, the susceptibility of polarized macrophages to Ag-specific CD8^+^ T cell killing is an unexplored issue. In order to shed light over this question, we induced macrophage polarization by culturing cells in the presence of IFN-γ, IL-4 or IL-10, and validated the cell phenotype by analyzing the expression of several surface markers by flow cytometry as reported previously^28^. Therefore, to assess the Ag-specific cytotoxicity, we adapted a method that allows the quantification of target cell death by 7-AAD incorporation and flow cytometry detection, as depicted in Fig. 2A^35^. To achieve that, macrophages were loaded with gamma-irradiated *M*. *tuberculosis*, or with a pool of antigens from Cytomegalovirus, Epstein Barr and Influenza (CEF) as a positive control for CD8^+^ T cell responses, since CEF encompass a set of antigens which are known to induce Ag-recall responses in human donors^36^. Afterwards, loaded macrophages were co-cultured with autologous CD8^+^ T cells expanded previously in the presence of the same antigens. Finally, target cell death was determined by 7-AAD exclusion within the macrophage gate by flow cytometry (Fig. 2B–D and Supplementary Fig. 2).

Our results show that both M1-polarized and M0 (GM-CSF-treated) macrophages were susceptible to *Mtb*-specific CD8^+^ T cell mediated killing, whereas alternatively activated macrophages (IL-4 or IL10) were not (Fig. 2C). Since basal macrophage death was dissimilar between IFN-γ and IL-4 polarizing treatments (Supplementary Fig. 3), we subtracted media values to Ag-specific cytotoxicity values and compare between treatments. Thus, we observed that M1-polarized macrophages were more susceptible than alternatively (IL-4) activated macrophages to *Mtb*-specific CD8^+^ T cell mediated killing (Fig. 2B,D). This increased susceptibility was independent of the nature of the antigen, as both *M*. *tuberculosis* (Fig. 2B,D) and CEF (Supplementary Fig. 4) antigens induced increased proportions of 7-AAD^+^ macrophages following co-culture. On the contrary, IL-4 or IL-10 polarization did not significantly increase CD8^+^ T cells’ mediated cytotoxicity over macrophages loaded with *Mtb* or CEF antigens (Fig. 2B–D and Supplementary Fig. 4). These results highlight the relevance of macrophage polarization on T cell cytotoxic responses during tuberculosis infection.

**Figure 3:**
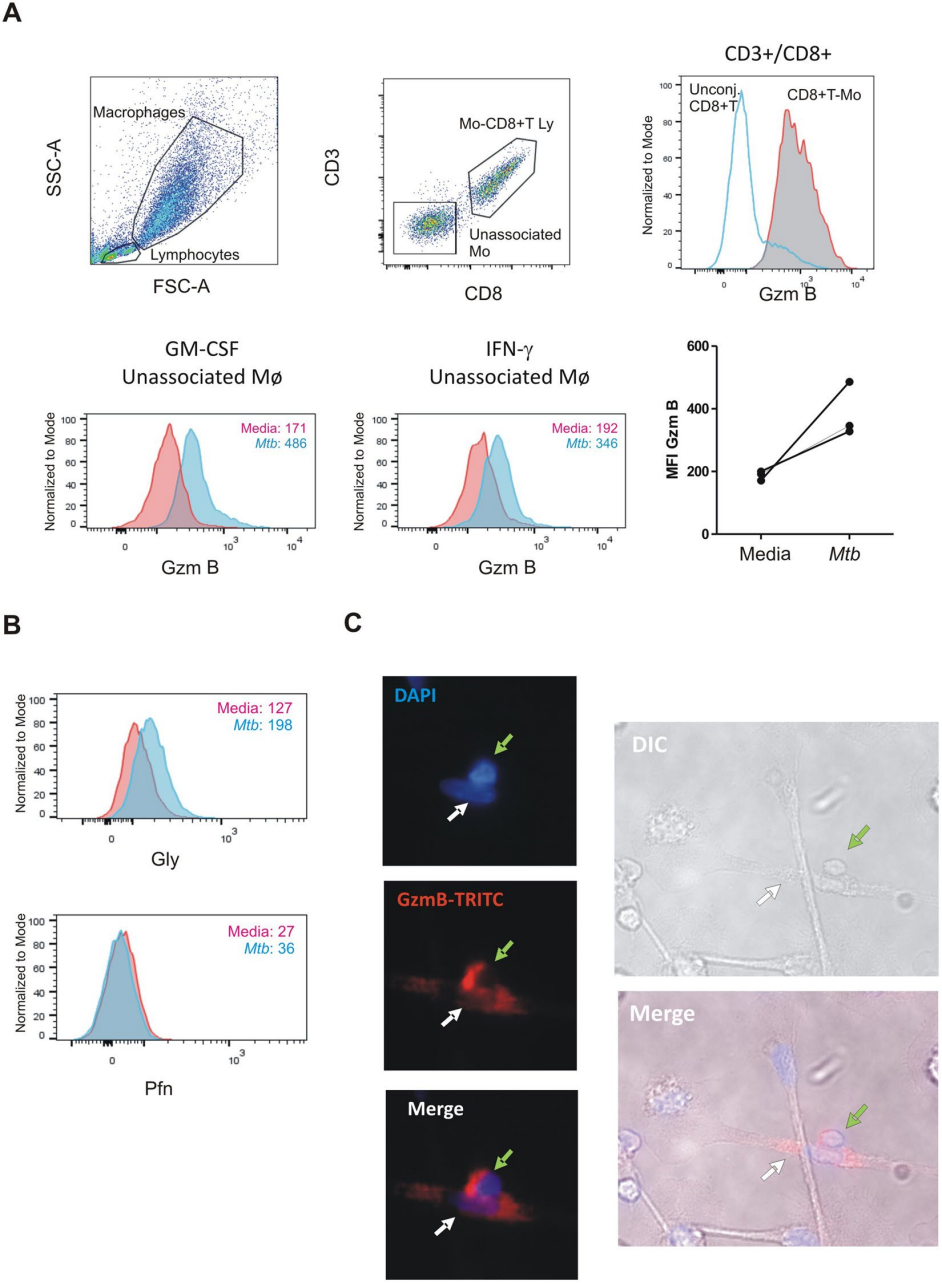
Transfer of effector molecules from cytotoxic lymphocytes to macrophages. (**A**) Representative Dot plots depicting the gating strategy for analyzing the transfer of Granzyme B (Grz B) from syngeneic CD8^+^ T cell effectors to macrophages loaded with *Mtb*. Figure shows from left to right (upper panel): FSC-A vs. SSC-A indicating macrophages and lymphocytes; CD3 vs. CD8 within the gate of macrophages, indicating double positive (Macrophages [Mo]-CD8^+^ T Ly cells complexes) and double negative (Unassociated Mo) events; Granzyme B (Gzm B) histogram, depicting Gzm B in unassociated lymphocytes (gated in the FSC/SSC graph, marked as Unconj. CD8^+^ T cells) vs. macrophage-associated lymphocytes, marked as CD8^+^T-Mo). Lower left panel: Gzm B in unassociated GM-CSF-treated macrophages from the co-cultures in media (pink) and *Mtb* (light blue). Inset numbers indicate median fluorescence intensity according to color code. Middle: Gzm B in unassociated IFN-γ-polarized macrophages from co-cultures in media (pink) and *Mtb* (light blue). Inset numbers indicate median fluorescence intensity according to color code. One representative example of three independent experiments performed in duplicate is shown. Right: Figure shows the MFI of intracellular Gzm B in un-conjugated macrophages before and after *Mtb* stimulation from 3 independent experiments, each one including one donor assayed by duplicate. (**B**) Effect of *Mtb*-stimulation on cytotoxic Granulysin (Gly, upper panel) and Perforin (Pfn, lower panel) transfer to macrophages. Histograms depict Gly and Pfn staining by flow cytometry in GM-CSF-treated macrophages loaded with *Mtb* antigens after 18 h co-culture with syngeneic CD8^+^ T cells in in media (pink) and *Mtb* (light blue). Inset numbers indicate median fluorescence intensity according to color code. (**C**) Gzm B staining in fixed 18 h Mø/CD8^+^ T cell co-cultures. Figure shows epifluorescence and DIC photographs of a macrophage (white arrow) and a CD8^+^ T cell (green arrow) in tight contact. One representative experiment of three independent experiments (one donor each) is shown.

CD8^+^ T cells can exert cytotoxicity through different mechanisms, including exocytosis of granules, death receptors like FAS, and TNF-α^37^. In this sense, we evinced CD8^+^ T lymphocyte-macrophage conjugates by both flow cytometry and fluorescence microscopy (Fig. 3A–C). In particular, Gzm content in such conjugates was greater compared to un-conjugated CD8^+^ T cells (Fig. 3A), therefore suggesting that conjugated CD8^+^ T cells were effector memory cells, as described previously^38^. In order to determine whether cytotoxic degranulation was involved in CD8^+^ T cell-induced cytotoxicity, we evaluated granzyme B (Gzm B), perforin (Pfn) and granulysin (Gly) transferred from CD8^+^ T cells to macrophages following co-culture. Thus, to evaluate the Gzm B transfer to macrophages and differentiate it from Gzm B expressed by cytotoxic lymphocytes, we studied the abundance of Gzm in un-conjugated macrophages, inferring that these effector molecules were indeed transferred from the cytotoxic lymphocytes. As depicted in Fig. 3A (lower panel), Gzm B content in both un-polarized and IFN-γ-polarized macrophages was increased after *M*. *tuberculosis* stimulation in comparison with un-stimulated cells. This effect was also observed in the transference of Gly, but it could not be detected in Pfn (Fig. 3B). Of note, the percentage of Pfn or Gly transfer was much lower than Gzm B transfer in all cases (Fig. 3A,B). Overall, these results show that CD8^+^ T cell cytotoxic degranulation is, at least, a mechanism involved in the cytotoxic process performed by antigen-specific lymphocytes.

### Effect of *M. tuberculosis* stimulation on macrophage polarization and PD-L1 expression

**Figure 4:**
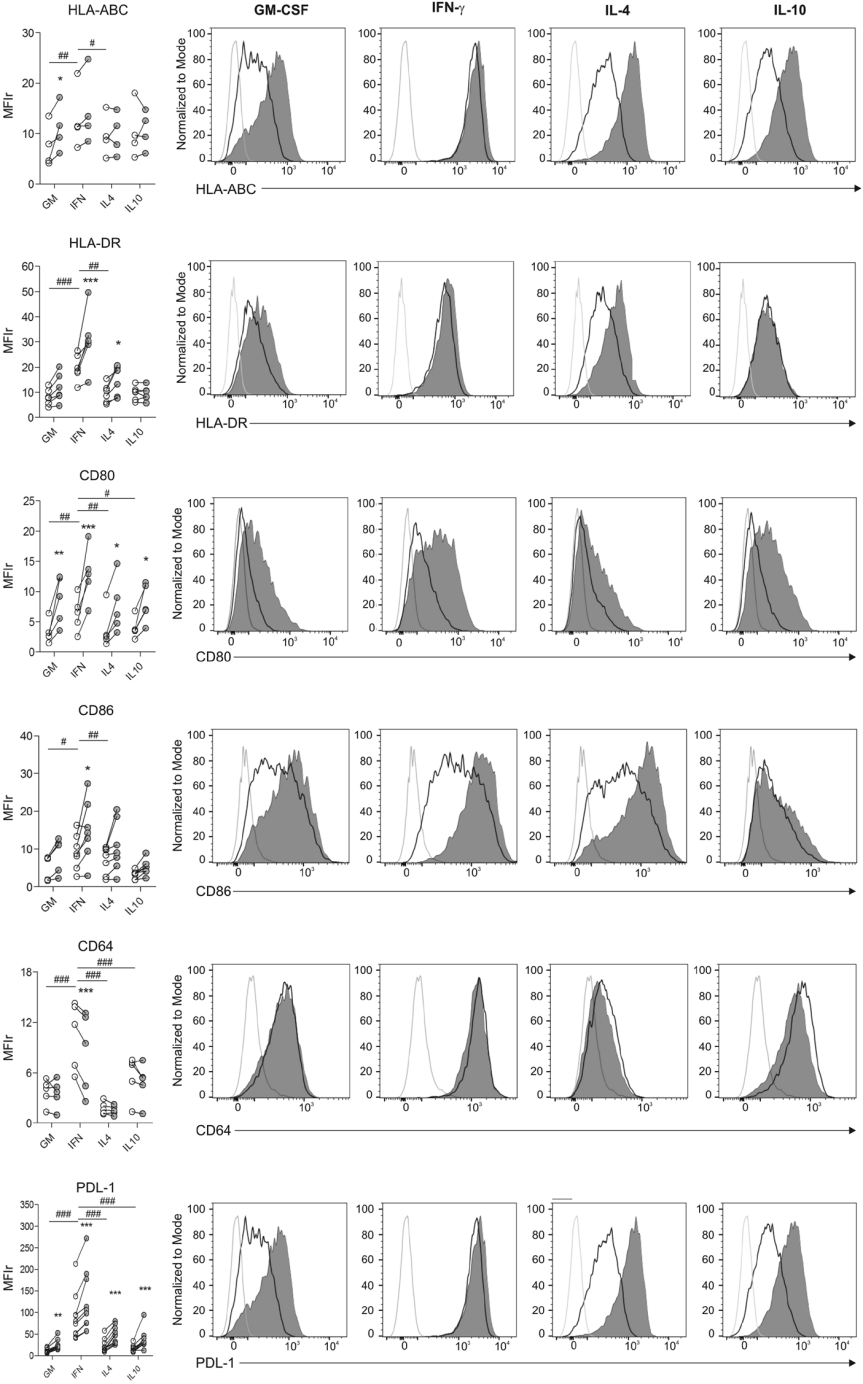
Study of the expression of HLA-ABC, HLA-DR, CD80, CD86, CD64 and PDL-1 on IFN-γ-, IL-4- and IL-10-polarized macrophages. Macrophages differentiated during 4–5 days with GM-CSF (30 ng/ml) were polarized in media containing GM-CSF alone or supplemented with IFN-γ (500 U/ml), IL-4 (10 ng/ml) or IL-10 (10 ng/ml) for 2 additional days. Left panels show data from 5 to 9 independent experiments performed in duplicate. Horizontal lines connect data from each individual experimental mean fluorescence intensity ratio (MFIr) from Media or *Mtb*-stimulated cells calculated as follows: MFIr = MFI of the indicated marker/MFI of the corresponding isotype control. Comparisons between polarizing conditions were done using ANOVA followed by Bonferroni posttest and were indicated with #. Alternatively, comparison between stimulated and un-stimulated conditions within polarized macrophages were performed with the Wilcoxon signed-rank test, and significances were depicted with asterisks. *or ^#^p < 0.05; **or ^##^p < 0.01; ***or ^###^p < 0.001. Flow cytometry histograms show data from a representative donor under the polarization conditions assayed, depicting for each marker the isotype control staining (open gray histogram), expression of the indicated marker on unstimulated (open black histograms) or alternatively, on stimulated macrophages (filled gray histograms).

**Figure 5:**
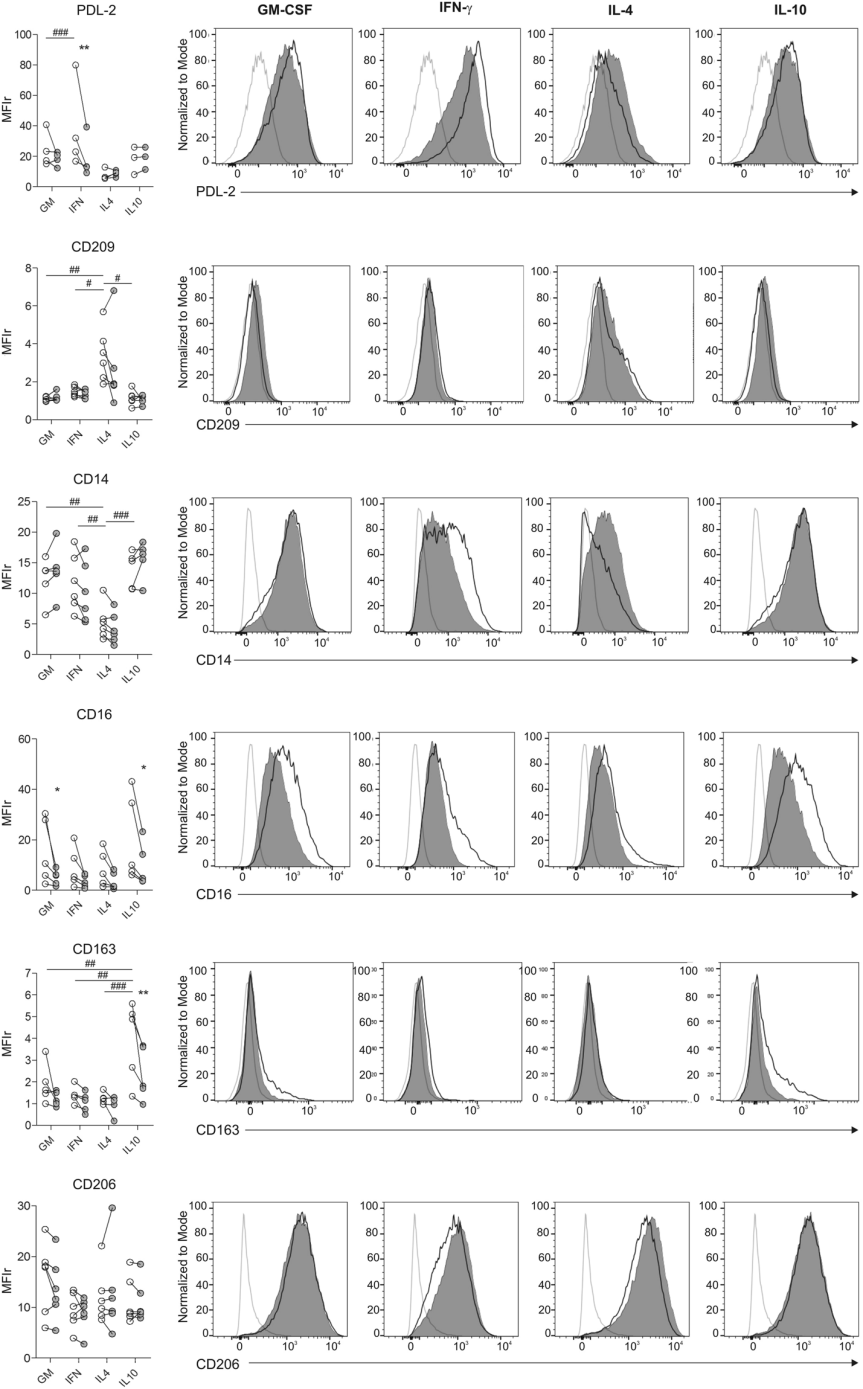
Study of the expression of PDL-2, CD209, CD14, CD16, CD163 and CD206 on IFN-γ-, IL-4- and IL-10-polarized macrophages. Macrophages were differentiated as described in Fig. 4. Left panels show data from 5 to 9 independent experiments performed in duplicate. Horizontal lines connect data from each individual experimental mean fluorescence intensity ratio (MFIr) from Media or *Mtb*-stimulated cells calculated as follows: MFIr = MFI of the indicated marker/MFI of the corresponding isotype control. Comparisons between polarizing conditions were done using ANOVA followed by Bonferroni posttest and were indicated with #. Alternatively, comparison between stimulated and un-stimulated conditions within polarized macrophages were performed with the Wilcoxon signed-rank test, and significances were depicted with asterisks. *or ^#^p < 0.05; **or ^##^p < 0.01; ***or ^###^p < 0.001. Flow cytometry histograms show data from a representative donor under the polarization conditions assayed, depicting for each marker the isotype control staining (open gray histogram), expression of the indicated marker on unstimulated (open black histograms) or alternatively, on stimulated macrophages (filled gray histograms).

As described in Fig. 2A, macrophage polarization was induced by activation of monocyte-derived macrophages with polarizing cytokines. As expected, we observed that activation of macrophages by IFN-γ significantly increased the expression of several molecules involved in antigen presentation and co-stimulation such as HLA-ABC, HLA-DR, CD80 and CD86, and also enhanced the expression of the high affinity IgG receptor involved in macrophage phagocytosis and activation, CD64 (Fc γ RI)^39^ (Fig. 4). Moreover, IFN-γ treatment significantly augmented PDL-1 (Fig. 4) and PDL-2 (Fig. 5) expression compared to other polarization stimuli. By contrast, IL-4 macrophages activation increased the expression of the M2 associated receptor CD209 (DC-SIGN)^40^ and down-modulated CD14, while IL-10 polarization increased surface levels of the M2c markers CD16 (Fc γ RIII) and CD163, both associated with tissue inflammation prevention^28,41^ (Fig. 5). Finally, the expression of the mannose receptor CD206 did not change under any polarization conditions (Fig. 5), supporting the notion posed previously that CD206 is not a specific marker for M2 polarization^28^.

Different components of *M*. *tuberculosis* can activate pattern recognition receptors (PRR) and therefore they can induce or modify macrophage polarization^42^. Accordingly, we aimed to investigate if gamma-irradiated mycobacteria could modify macrophage polarization. We observed that *M*. *tuberculosis* stimulation increased HLA-ABC expression on un-polarized macrophages, HLA-DR on IFN-γ - and IL-4 - activated macrophages and CD86 on IFN-γ - activated macrophages. On the other hand, *M*. *tuberculosis* stimulation decreased the surface protein levels of CD64 on IFN-γ-activated macrophages, of CD16 both on GM-CSF - and IL-10 - activated macrophages and of PDL-2 on IFN-γ-activated cells (Figs 4 and 5). Notably, among all the markers analyzed, only CD80 and PD-L1 were significantly increased by *M*. *tuberculosis* stimulation independently of the polarization state (Fig. 4). On the contrary, CEF stimulation had no effect on the expression of neither PD-L1 nor PD-L2 regardless of the activation program (Supplementary Fig. 5). Therefore, *M*. *tuberculosis* stimulation exerted a significant modulatory effect over M2c macrophage polarization by down-regulating both CD16 and CD163. Moreover, it significantly enhanced the expression of CD80 and PD-L1, two molecules involved in the regulation of CD8^+^ T cell activation.

Based on this, since the balance between co-stimulatory and inhibitory signals may determine the magnitude of T cell activation^29^, we decided to compare the effect of macrophage polarization and *M*. *tuberculosis* stimulation over PD-L1 and CD80 expression. We therefore observed that the effect of IFN-γ on PD-L1 expression over GM-CSF was about four times greater than the increase induced by IFN-γ on CD80 levels, in contrast to other polarization conditions, where both receptors increased their expression in similar proportions (Supplementary Fig. 6A). Additionally, the combined effect of IFN-γ plus *M*. *tuberculosis* stimulation increased two-fold on PD-L1 expression compared to CD80 over unstimulated conditions -GM-CSF- (Supplementary Fig. 6B).

Based on this, we propose that M1 macrophages and more importantly, the M1 macrophages exposed to *M*. *tuberculosis* could exert an inhibition on T cell responses through PD-1/PD-L1 interactions.

### Effect of PD-L1 blockade on macrophage killing by cytotoxic CD8^+^ T cells

**Figure 6:**
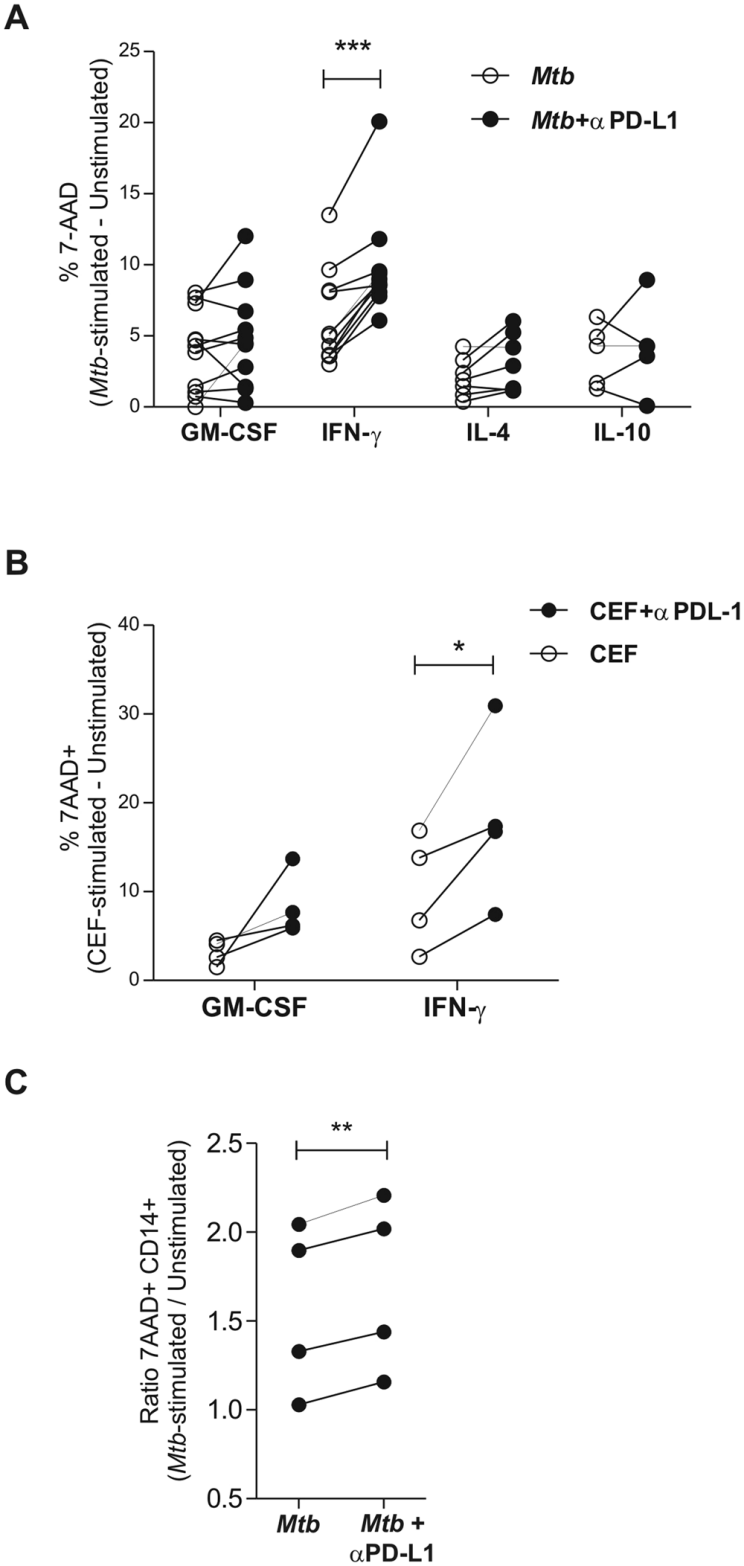
Effect of PD-L1 blockade on CD8^+^ T cell-mediated cytotoxicity. Macrophages were differentiated as described above and afterwards were loaded with. (**A**) *Mtb* (10 μg/ml) or (**B**) CEF (1 ng/ml) plus IL-2, and co-cultured during 18 h with syngeneic CD8^+^ T cells (previously expanded with the same antigens) at a 2:1 CD8^+^ T cell:Mø ratio, in the presence or absence of anti-PD-L1 blocking antibodies. Horizontal lines connect the results from each individual experiment performed in duplicate showing the % of 7-AAD^+^ macrophages treated with PDL-1 blocking antibodies or left untreated. ***p < 0.001 and *p** < **0.05 by ANOVA followed by Bonferroni post-test. Media values were subtracted and negative values were set to 0. (**C)** CD14^+^ and CD8^+^ cells were isolated from tuberculous pleural effusion samples by positive and negative selection, respectively. Afterwards, CD14^+^ macrophages were loaded with gamma-irradiated *Mtb* (10 μg/ml), extensively washed and co-cultured for 18 h with autologous CD8^+^ T cells in the presence or absence of anti-PDL-1 blocking antibodies. Finally, macrophage death was determined by 7-AAD incorporation by flow cytometry. The ratio between 7-AAD^+^ *Mtb*-treated macrophages and media values were calculated for each donor and horizontal lines connect data from each individual. **p < 0.01, Student *t* Test for paired samples.

Taking into account that i) PD-Ls were highly expressed in PE cells from TB patients; ii) M1 macrophages were more susceptible to cytotoxic killing by CD8^+^ T cells, and iii) that PD-1 pathway blockade enhanced cytotoxicity and IFN-γ production by PEMC from TB patients as observed elsewhere^22^, we aimed to study the modulatory role of PD-1/PD-Ls pathway on CD8^+^ T cell-driven antigen-specific cytotoxicity. To achieve this, we performed *in vitro* cytotoxic assays in the presence of anti-PD-Ls blocking antibodies, as depicted in Fig. 2A. Thus, we observed that PD-L1 blocking significantly increased CD8^+^ T cell cytotoxicity against IFN-γ-activated macrophages loaded both with *M*. *tuberculosis* (Fig. 6A) or CEF antigens (Fig. 6B). On the contrary, PD-L1 blocking had no effect on *in vitro* cytotoxicity against either un-polarized macrophages, IL-4- or IL-10 treated macrophages (Fig. 6A,B). Moreover, PD-L2 blockade did not have any effect on antigen specific cytotoxicity as well (Supplementary Fig. 7, and data not shown). Thus, even when IFN-γ -activated macrophages are more susceptible to *M*. *tuberculosis*-specific CD8^+^ T cell cytotoxicity, these macrophages can inhibit CD8^+^ T cell-mediated cytotoxicity by increasing PDL-1 expression. Finally, we aimed to study the role of PD-1 pathway over CD8^+^ T cell-driven antigen-specific cytotoxicity in the context of human tuberculous pleurisy. To that, CD14^+^ macrophages from TB pleural effusion samples were loaded with gamma-irradiated *Mtb*, extensively washed and co-cultured with autologous CD8^+^ T cells in the presence or absence of anti-PD-L1 blocking antibodies. We then observed that PD-L1 blocking enhanced *Mtb*-specific CD8^+^ T cell mediated killing of CD14^+^ PE cells (Fig. 6C), therefore highlighting the relevance of PD-1 pathway in CD8^+^ T cell-induced macrophage death during human tuberculosis.

Overall, these results indicate that PD-1/PD-L1 pathway impedes *M*. *tuberculosis*-specific cytotoxicity against macrophages polarized by IFN-γ to an M1 profile.

## Discussion

Control of *M*. *tuberculosis* infection depends on both innate and adaptive immune responses. Moreover, effective killing of infected macrophages is considered a major strategy for bacterial control. In this context, we hypothesized that macrophage polarization along with PD-1/PDL-1 pathway can shape the function of cytotoxic *Mtb*-specific CD8 T^+^ cells. Our results show that PD-1 and PDL-1 molecules were highly expressed at the site of infection in TB samples concomitantly with high IFN-γ levels at the site of the tuberculous pleurisy, and their expression positively correlated with T-cell cytotoxic functions in human tuberculosis. Moreover, and according to our *in vitro* experiments, we observed that M1 macrophage polarization induced the strongest cytotoxic Ag-specific response compared to M2 polarization. Paradoxically, IFN-γ stimulation increased PD-L1 expression in macrophages and cytotoxic killing was enhanced when the PD-1 /PDL-1 interaction was abolished. Also, blocking PD-L1 pathway only enhanced Ag- specific cytotoxicity in M1 macrophages, therefore unraveling the relevance of this interaction in the context of M1 polarization. Finally, the relevance of PD-1 /PD-L1 pathway over these processes was unraveled when PDL-1 blocking increased *Mtb*-specific cytotoxic killing of CD14^+^ targets from human PE samples.

Our observations indicate a relationship between CD8^+^ T cell cytotoxic potential (measured using CD107a/b expression as a surrogate marker for degranulation^43^) and PD-1 expression on CD8^+^ T cells and/or PDL-1 expression on PE CD14^+^ cells. Previously, we demonstrated that blockage of PD-1/PDL-1 signaling pathway could enhance cytotoxic degranulation of CD8^+^ T cells from TB pleural effusion samples, therefore suggesting a protective role for PD-1 pathway by limiting tissue damage^22^. The results presented in this work are in line with the above, highlighting the relevance of PD-1/PDL-1 pathway in this process which led us to conclude that PDL-1 blockade could increase macrophage susceptibility to death by enhancing the cytotoxic potential of CD8^+^ T cells.

Our results indicate that the activation of macrophages to an M1 profile by IFN-γ increased its susceptibility to lysis by *M*. *tuberculosis*- specific CD8^+^ T lymphocytes compared to non-polarized macrophages (GM-CSF-treated) or activated to M2 type profiles (IL-10 or IL-4-treated). Remarkably, the cytotoxic mechanism involved, at least, the transfer of lytic molecules to macrophages, as we observed that macrophages expressed Gzm B and Gly after co-culture with CD8^+^ lymphocytes. The fact that those CD8^+^ T cells with the highest cytotoxic molecule expression levels were those associated with polarized macrophages suggests that these CD8^+^ T cells were probably effector cells, as described elsewhere^38,44^.

This study also demonstrates that *Mtb*-stimulation did not induce any changes in the overall phenotype of IFN-γ or IL-4-polarized macrophages, as shown in Figs 4 and 5. Instead, *Mtb* stimulation induced a down-modulation of CD16 and CD163 expression, two markers for M2c differentiation, on IL-10-treated macrophages. This data acquires relevance in the context of an infection such as tuberculosis, since IL-10 is a cytokine dramatically enhanced at the site of infection^45^ and although we could not detect any changes *in vitro*, it could modify macrophage susceptibility to lysis by CD8^+^ T cell effectors at the site of infection, as suggested by others^14^.

Our experimental model included the use of two different antigens. On the one hand, gamma-irradiated *M*. *tuberculosis* is a particulate antigen which is phagocytized by macrophages^42^. On the other hand, CEF consist of a pool of short peptides from usual viral infections, which do not need to be processed in order to be presented in the context of HLA-I molecules^46^. The fact that macrophage polarization by IFN-γ increased CD8^+^ T cells- cytotoxicity to both CEF and *Mtb*-loaded macrophages in a similar extent, suggests that this effect is not related to changes in antigen processing pathways. Alternatively, this increased susceptibility could be related to an increased expression of HLA class I and costimulatory molecules, as shown here. In line with this, two previous reports showed that M2-polarization is related to inhibition of CD8^+^ T cell cytotoxic responses, and that the addition of IFN-γ can revert this inhibition^14,47^ by increasing CD86 expression^14^, highlighting the relevance of costimulatory molecules in this process.

As demonstrated previously, the balance between the activation and the inhibitory signals defines the ultimate result on T cell activation and function. Our results show that macrophage activation by IFN-γ increased PD-L1 expression by 10 fold and that *Mtb* stimulation further increased it. Intriguingly, the PD-L1 increment was particularly high compared to other activating costimulatory molecules like CD80 or CD86. These results are in consonance with the increment in PD-L1 expression by CD14^+^ cells from TB pleural effusions shown in Fig. 1. Additionally, our results demonstrate that the PD-L1 blockade increased cytotoxicity to M1-macrophages *in vitro*, but not to M2-macrophages, and that PD-L2 blockade had no effect on cytotoxicity. Therefore, these data in addition to the observations showing that PD-L1 blocking enhanced *Mtb*-specific CD8^+^ T cell mediated killing of CD14^+^ PE cells (Fig. 6C) and the high IFN-γ concentrations in the pleural compartment (Supplementary Fig. 1) support the hypothesis indicating that the increment in PD-L1 expression by IFN-γ activation may have a protective role by preventing the killing of macrophages. Conversely, PD-L1 induction by *Mtb* is probably an immune evasion mechanism that hinders the killing of infected cells.

Of note, PD-1 expression on CD8^+^ T cells was highly increased in TB pleural effusions and also it was limited to a T_CM_ phenotype^33^ (and Fig. 1), indicating that cytotoxic responses are regulated by this pathway during TB pleurisy. Our observations suggest, as detected previously in HIV-TB^8^ and HIV^+^ ^33^ individuals, an arrest of exhausted cells on a less differentiated and poor functional phenotype. In this line, Buggert *et*. *al* documented a CD8^+^ PD-1^+^ T-bet^low^/Eomes^high^ T_TE_ population in HIV^+^ patients with exhausted characteristics and arrested in this differentiation state^33^. Finally, our results are also in line with other authors, who observed a prominent T central memory population and a decrease in terminally-differentiated T cells specific for Ag85A in HIV-negative tuberculosis patients, which was reversed following anti-tuberculosis therapy^48^.

While PD-1 treatment was found to be extremely toxic in animal models of TB infection, its deleterious effects were mainly related to its action over CD4^+^ T cells^23,24^. Based on our results, it will be of interest to evaluate the effect of CD8^+^ T cell-targeted anti-PD-1 treatments during TB infection focusing on new approaches, such as the one described to enhance human Ag-specific cytotoxic-T lymphocytes by performing PD-1 gene knockouts^20^.

In sum, our results are in line with a model in which, upon recognition of *Mtb*-infected cells, innate and adaptive immune lymphocytes secrete IFN-γ, leading to the activation of macrophages to a M1 profile, therefore increasing macrophage susceptibility to CD8^+^ T cell-mediated cytotoxicity. This effect could be associated with the increment in HLA class I and costimulatory molecules expression. In parallel, the same stimulus could lead to a higher PD-L1 expression, which combined with the increased PD-1 expression in CD8^+^ T cells could inhibit *Mtb*-specific CD8^+^ T cell cytotoxicity. The balance between these stimulatory and inhibitory signals will define the fate of the target cell, and finally delineate the resolution of the infection.

## Electronic supplementary material

Supplementary  information
