# Supplementary material for: PD-1/PD-L1 Pathway Modulates Macrophage Susceptibility to Mycobacterium tuberculosis Specific CD8+ T cell Induced Death
Source: Sci Rep. 2019 Jan 17;9:187. doi: 10.1038/s41598-018-36403-2 (PMC6336852; doi:10.1038/s41598-018-36403-2)
Supplement: Supplementary file 1 — Supplementary information [file 41598_2018_36403_MOESM1_ESM.pdf]

Supplementary Material for

PD-1 / PD-L1 Pathway Modulates Macrophage Susceptibility to *Mycobacterium tuberculosis* Specific CD8$^+$ T cell Induced Death.

Guadalupe Verónica Suarez, Claudia del Carmen Melucci Ganzarain, María Belén Vecchione, César Ariel Trifone, José Luis Marín Franco, Melanie Genoula, Eduardo José Moraña, Luciana Balboa and Maria Florencia Quiroga.

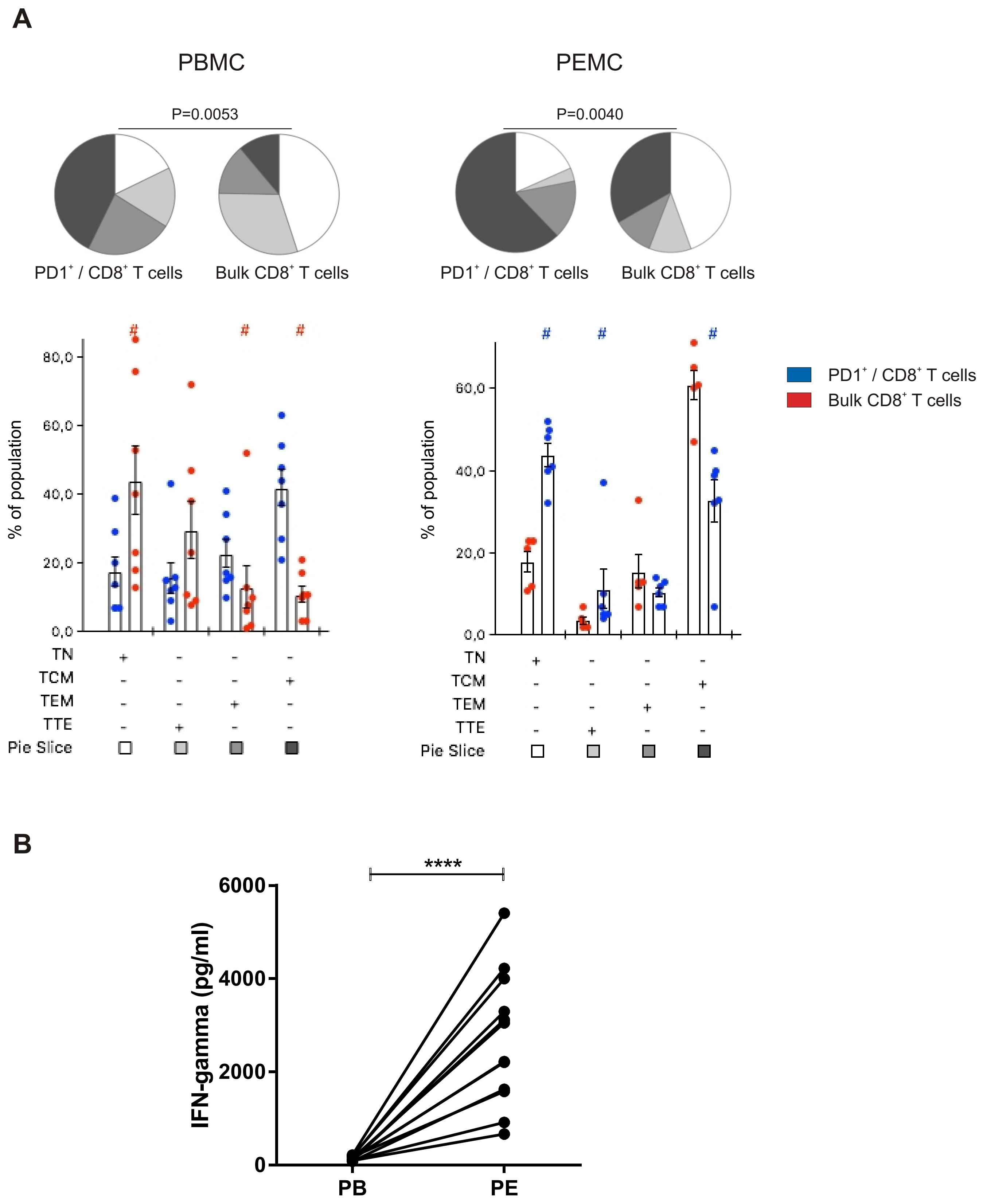

Supplementary Figure 1. A. Analysis of the memory / effectory distribution between PD1+ and bulk CD8+ T cells from peripheral blood (PBMC, left) and pleural effusion mononuclear cells (PEMC, right). Bottom: pie charts summarize the data and each slice corresponds to the mean proportion of CD3+/CD8+/PD-1+ or CD3+/CD8+ cells for each phenotype. Bar graph represents possible phenotypes, which are shown on the x-axis whereas percentages of distinct T-cell subsets within CD3+/CD8+/PD-1+ or CD3+/CD8+ cells are shown on the y-axis. Horizontal lines represent the median range and each point represents an individual subject; asterisks indicate a significant difference between groups; #p < 0.05. Comparisons of phenotype distribution were performed using the partial permutation test as described in Ref. 32 and the Kruskal Wallis test followed by Dunn's multiple comparisons posttest. **B.** IFN-gamma assessment comparing matched Pleural Effusion and Peripheral Blood samples from TB patients. Horizontal lines connect data from each individual. ****, *p*<0.0001, Mann Whitney test.

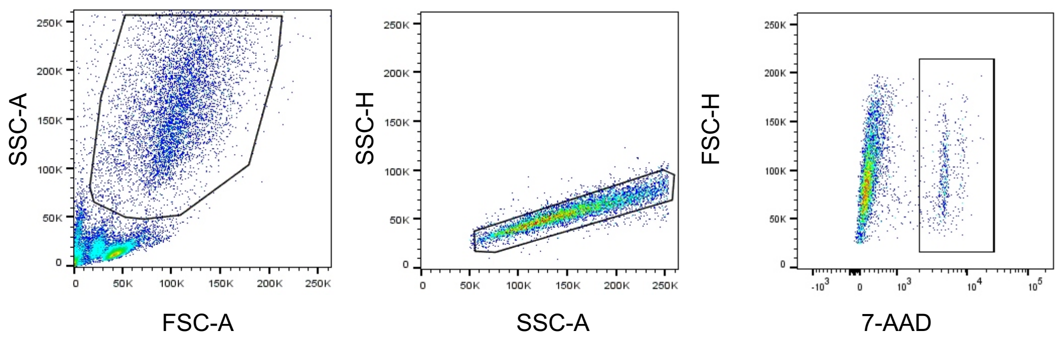

Supplementary Figure 2. Gating strategy for the determination of 7-AAD macrophage incorporation after co-culture with CD8+ T cells. Monocyte derived macrophages (Mø) were differentiated from peripheral blood by culturing monocytes as described in *Methods* section. Then, Mø were cultured with *M. tuberculosis* (10 µg/ml) or left in cell culture media. After that, Mø were co-cultured for 18 h with syngeneic CD8+ T cells previously expanded with *M. tuberculosis* (*Mtb*, 10 µg/ml). Finally, Mø death was determined by 7-AAD incorporation by flow cytometry. Representative dot plots show from left to right: FSC-A vs. SSC-A; SSC-A vs. SSC-H (to discriminate signlets) and 7-AAD vs. FSC-A graphs from representative co-cultures of CD8+ T cells and Mø (2:1 ratio) loaded with *Mtb*.

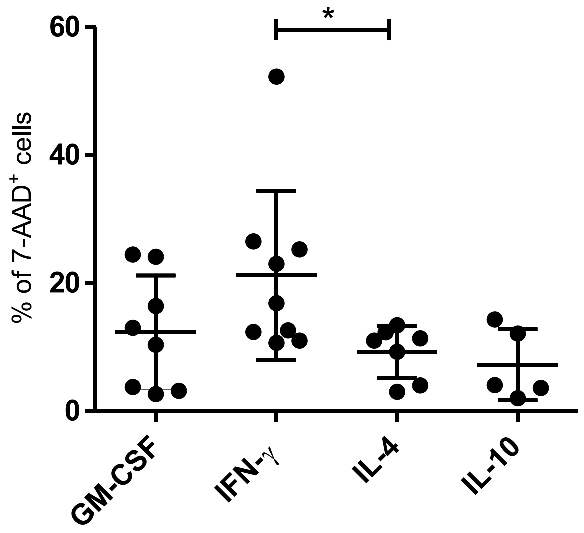

Supplementary Figure 3. Effect of macrophage polarization on *in vitro* macrophage basal death. Monocyte derived macrophages (Mø) were differentiated from peripheral blood by culturing monocytes with GM-CSF (30 ng/ml) for 6-7 days alone or supplemented with IFN-γ (500 U/ml), IL-4 (10 ng/ml) or IL-10 (10 ng/ml) for 2 additional days. Then, Mø were cultured with media for the remaining 18 h with syngeneic CD8+ T cells previously expanded with gamma-irradiated *Mtb*. Finally, Mø death was determined by 7-AAD incorporation by flow cytometry. Figure shows the % of 7-AAD+ Mø from 8 independent experiments, each one including one donor assayed by duplicate. * $p<0{,}05$, by ANOVA followed by Bonferroni posttest.

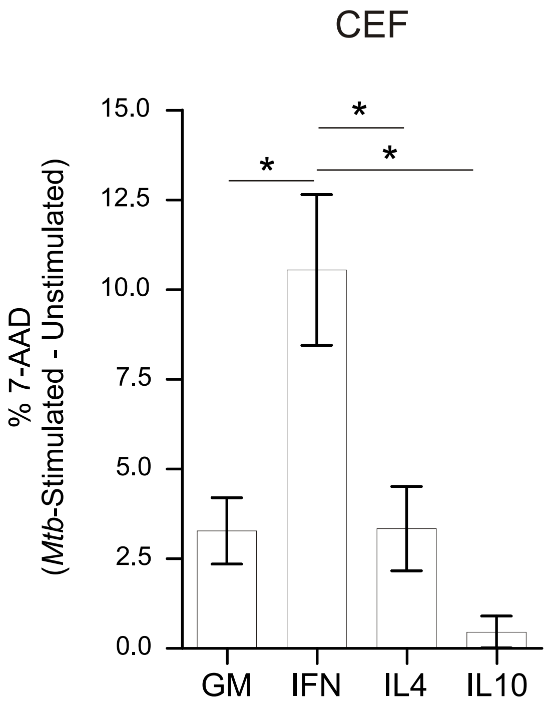

Supplementary Figure 4. Effect of macrophage polarization on in vitro CD8+ T cell-mediated cytotoxicity. Monocyte derived macrophages (Mø) were differentiated from peripheral blood by culturing monocytes with GM-CSF (30 ng/ml) for 6-7 days alone or supplemented with IFN-γ (500 U/ml), IL-4 (10 ng/ml) or IL-10 (10 ng/ml) for 2 additional days. Then, Mø were cultured with CEF (5 μg/ml) as described in Methods. After that, Mø were co-cultured for 18 h with syngeneic CD8+ T cells previously expanded with CEF (1 μg/ml) + IL-2 (100 UI/ml). Finally, Mø death was determined by 7-AAD incorporation by flow cytometry. Figure shows the % of 7-AAD+ Mø from 5 independent experiments, each one including one donor assayed by duplicate. Media values were subtracted and negative values were set to 0. * p<0,05, ** p<0,01; *** p<0,001 by ANOVA followed by Bonferroni posttest.

Supplementary Figure 5

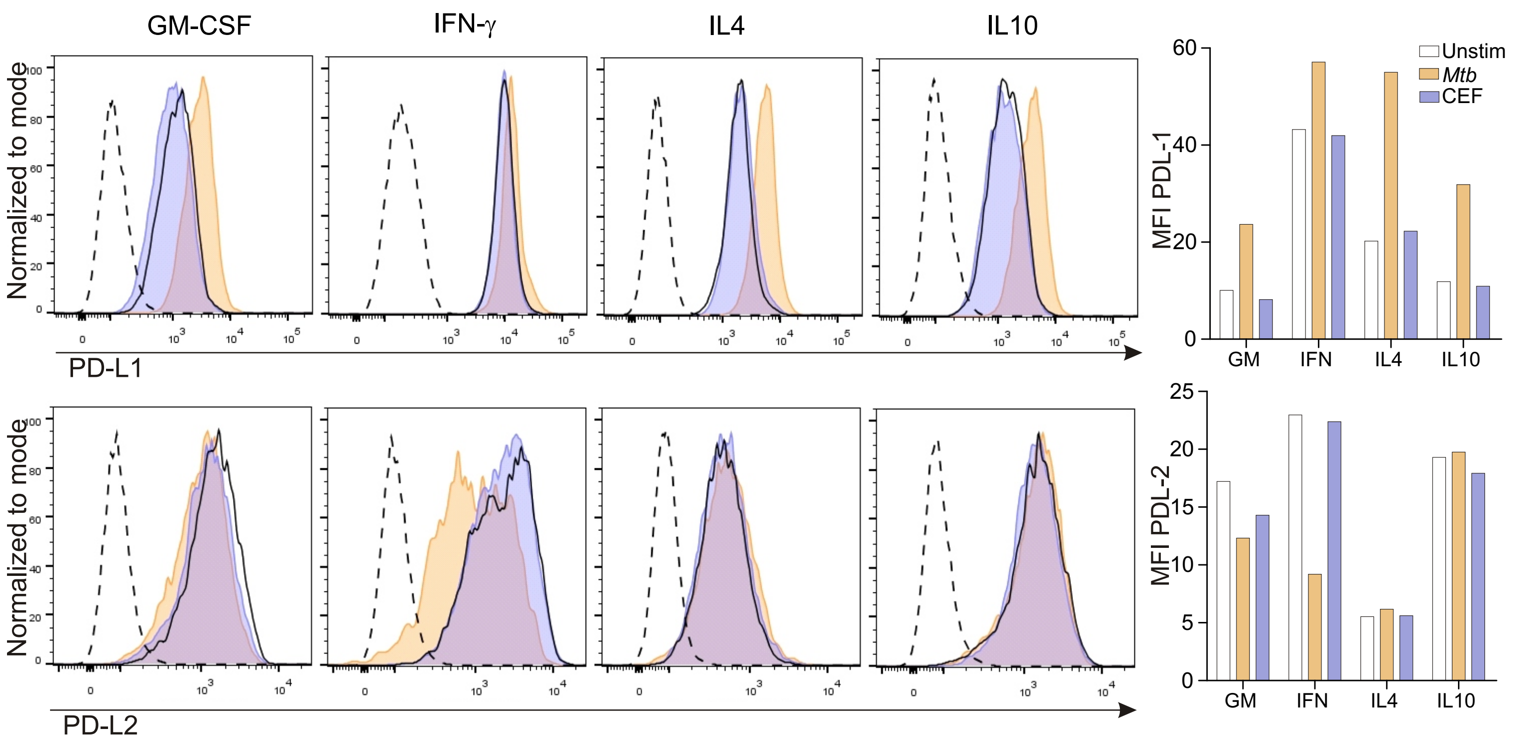

Supplementary Figure 5. Effect of the stimulation with *M. tuberculosis* or CEF over the expression of PD-L1 or PD-L2 in macrophages polarized with IFN-γ, IL-4 or IL-10. Mø differentiated during 4-5 days with GM-CSF (30 ng/ml) were polarized in media containing GM-CSF alone or supplemented with IFN-γ (500 U/ml), IL-4 (10 ng/ml) or IL-10 (10 ng/ml) for 2 additional days. Then, Mø were incubated with *M. tuberculosis* (10 ug/ml) or CEF (5 ng/ml) for 18 h and the expression PD-L1 and PD-L2 was evaluated by flow cytometry. Figure shows histograms (left) and mean fluorescence intensity (MFI) of the expression of PD-L1 (top) and PD-L2 (bottom) of a representative experiment (one donor in duplicate) out of 3.
Histograms depict isotype control staining (dashed lines), PDL-1 or PDL-2 staining from cells in media (Unstimulated, solid line), and PDL-1 or PDL-2 expression on *Mtb*- (orange) or CEF- (blue) stimulated macrophages.

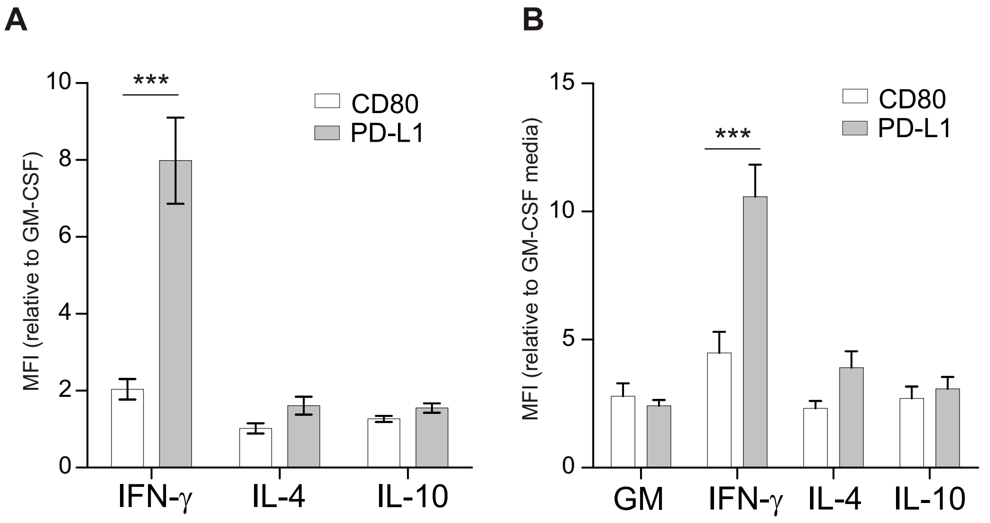

Supplentary Figure 6. Comparison of the effect of polarization and *M. tuberculosis* stimulation over the expression of CD80 and PD-L1 in Mø. Values of the mean fluorescence intensity (MFI) of CD80 and PD-L1 from Figure 4 were relativized and compared. A. Figure shows the relative increment of the expression of CD80 and PD-L1 induced by Mø polarization with FN-γ, IL-4 o IL-10 over non polarized Mø. For each donor, the ratio between the expression of each molecule in polarized Mø over non polarized Mø (GM-CSF) was calculated. B. Relative increment of the expression of CD80 and PD-L1 induced by both polarization and *M. tuberculosis* stimulation. For each donor, the ratio between the expression of each molecule in polarized Mø stimulated with *M. tuberculosis* over non polarized Mø (GM-CSF) in media was calculated. Figure shows media ± SEM from between 3 and 6 independent experiments with between 3 and 10 donors. *** p<0,001 by two way ANOVA followed by Bonferroni post test.

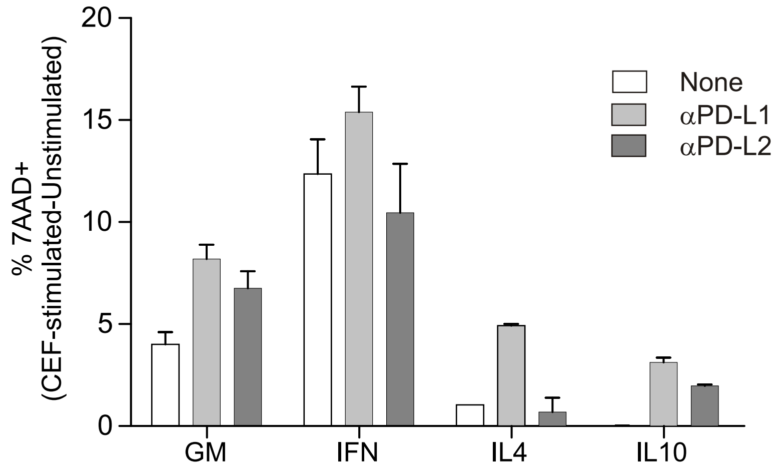

Supplementary Figure 7. Effect of PD-L1/PD-L2 blockade on CD8+ T cell-mediated in vitro cytotoxicity. Mø differentiated during 4-5 days with GM-CSF (30 ng/ml) were polarized in media containing GM-CSF alone or supplemented with IFN-γ (500 U/ml), IL-4 (10 ng/ml), IL-10 (10 ng/ml) or IFN-γ (500 U/ml) for 2 additional days. Then, Mø were loaded with CEF (5 μg/ml) and were co-cultured during 18 h with syngeneic CD8+ T cells previously stimulated with CEF, at a 2:1 CD8+ T cell:Mø ratio in the presence or absence of anti-PD-L1 or anti-PD-L2 blocking antibodies. Figure shows mean ± SEM of the % of 7-AAD+ Mø from one representative experiment of 4. Media values were subtracted and negative values were set to 0.
